# Click-PEGylation – A mobility shift approach to assess the redox state of cysteines in candidate proteins

**DOI:** 10.1016/j.freeradbiomed.2017.03.037

**Published:** 2017-07

**Authors:** Lucie A.G. van Leeuwen, Elizabeth C. Hinchy, Michael P. Murphy, Ellen L. Robb, Helena M. Cochemé

**Affiliations:** a MRC London Institute of Medical Sciences, Du Cane Road, London W12 0NN, UK; b Institute of Clinical Sciences, Imperial College London, Hammersmith Hospital Campus, Du Cane Road, London W12 0NN, UK; c MRC Mitochondrial Biology Unit, University of Cambridge, Wellcome Trust/MRC Building, Cambridge Biomedical Campus, Hills Road, Cambridge CB2 0XY, UK

**Keywords:** DTT, dithiothreitol, GAPDH, glyceraldehyde-3-phosphate dehydrogenase, NEM, *N*-ethylmaleimide, PEG, polyethylene glycol, TCEP, tris(2-carboxyethyl)phosphine, Redox, Cysteine, Thiol, Click chemistry, Mobility shift, PEGylation

## Abstract

The redox state of cysteine thiols is critical for protein function. Whereas cysteines play an important role in the maintenance of protein structure through the formation of internal disulfides, their nucleophilic thiol groups can become oxidatively modified in response to diverse redox challenges and thereby function in signalling and antioxidant defences. These oxidative modifications occur in response to a range of agents and stimuli, and can lead to the existence of multiple redox states for a given protein. To assess the role(s) of a protein in redox signalling and antioxidant defence, it is thus vital to be able to assess which of the multiple thiol redox states are present and to investigate how these alter under different conditions. While this can be done by a range of mass spectrometric-based methods, these are time-consuming, costly, and best suited to study abundant proteins or to perform an unbiased proteomic screen. One approach that can facilitate a targeted assessment of candidate proteins, as well as proteins that are low in abundance or proteomically challenging, is by electrophoretic mobility shift assays. Redox-modified cysteine residues are selectively tagged with a large group, such as a polyethylene glycol (PEG) polymer, and then the proteins are separated by electrophoresis followed by immunoblotting, which allows the inference of redox changes based on band shifts. However, the applicability of this method has been impaired by the difficulty of cleanly modifying protein thiols by large PEG reagents. To establish a more robust method for redox-selective PEGylation, we have utilised a Click chemistry approach, where free thiol groups are first labelled with a reagent modified to contain an alkyne moiety, which is subsequently Click-reacted with a PEG molecule containing a complementary azide function. This strategy can be adapted to study reversibly reduced or oxidised cysteines. Separation of the thiol labelling step from the PEG conjugation greatly facilitates the fidelity and flexibility of this approach. Here we show how the Click-PEGylation technique can be used to interrogate the redox state of proteins.

## Highlights

• Click-PEGylation is a mobility shift assay to assess protein thiol redox state.
• Separating thiol labelling from PEGylation by Click chemistry allows flexibility.
• Both reduced and oxidised thiols are assessed using Click-PEG_*red*_ and Click-PEG_*ox*_.
• Multiple targets can be interrogated in a single sample using different antibodies.

## Graphical abstract

**Figure:**
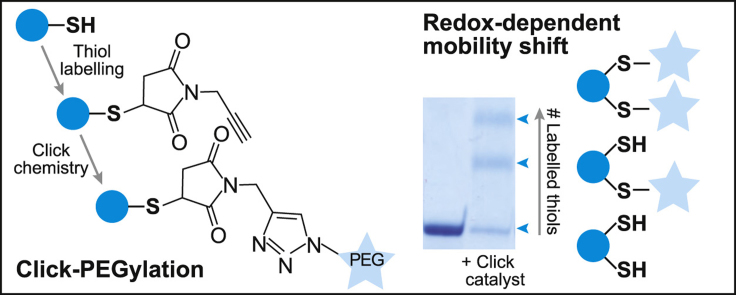

## 1 Introduction

Cysteine residues in proteins contain reactive thiol groups, whose redox state is a critical mediator of protein function. Under normal physiological conditions, the majority of exposed thiol groups are maintained in a reduced state [1], [2]. However, fluctuations in cellular redox state can lead to a range of oxidative thiol modifications, including the formation of disulfide bonds, *S*-sulfenylation, *S*-nitrosation, *S*-acetylation or *S*-glutathionylation [3], [4]. These oxidative modifications can affect the activity, binding interactions, lifetime or localisation of a protein [5]. Importantly, the majority of these post-translational redox modifications are reversible, therefore capable of functioning as a signalling mechanism, as well as in the response of the cell to oxidative stress [6]. Thiol groups can become irreversibly oxidised under conditions of oxidative stress, often leading to degradation of the affected proteins. Compared to other amino acids, cysteines are under-represented in proteins due to their high reactivity and either tend to be extremely highly, or very poorly, conserved [7]. However, not all cysteines are equally susceptible to oxidative modification. For instance, solvent-exposed cysteines are more likely to be redox-sensitive than cysteines buried within the protein structure [8]. Since multiple redox-reactive cysteine residues with different susceptibilities to oxidation can be present within a protein, many redox states are possible for a single protein. Thus, it is important to be able to identify and quantify the changes in these protein thiols under various biological conditions.

A number of methods are available to assess the redox state of protein thiols, the most quantitative of which are redox proteomic approaches utilising mass spectrometry, such as OxICAT [9], [10]. These unbiased approaches can both identify the cysteine residues affected and quantify the extent of oxidation. However, these untargeted screens are time-consuming and coverage is often biased towards abundant proteins. In addition, detection of cysteine residues by mass spectrometry can be complicated by their location within regions that are challenging to access using currently available proteases. Therefore, candidate protein approaches based on antibody binding are often used in parallel to enable low abundance proteins to be interrogated, or to obtain a more complete redox profile of the protein of interest. Typically, the redox-altered protein thiols are selectively tagged to a large group, such as a polyethylene glycol (PEG) moiety connected to a maleimide compound [11]. Maleimide compounds react selectively and irreversibly with reduced thiol groups through the formation of a thioether bond. Samples can then be separated by electrophoresis, and probed with an antibody against the protein of interest, avoiding the need to purify proteins prior to analysis. PEGylated cysteines will cause a mobility shift, and the result will typically show a series of bands corresponding to different redox forms of the protein interrogated. Providing an appropriate antibody is available, the extent of the redox modification for even low abundance proteins can be quantified quickly and cheaply. However, a limitation of this approach is that the reaction of a cysteine residue with a large (typically ~2–5 kDa) activated PEG moiety can be slow, leading to incomplete reaction with protein thiols.

**Fig. 1:**
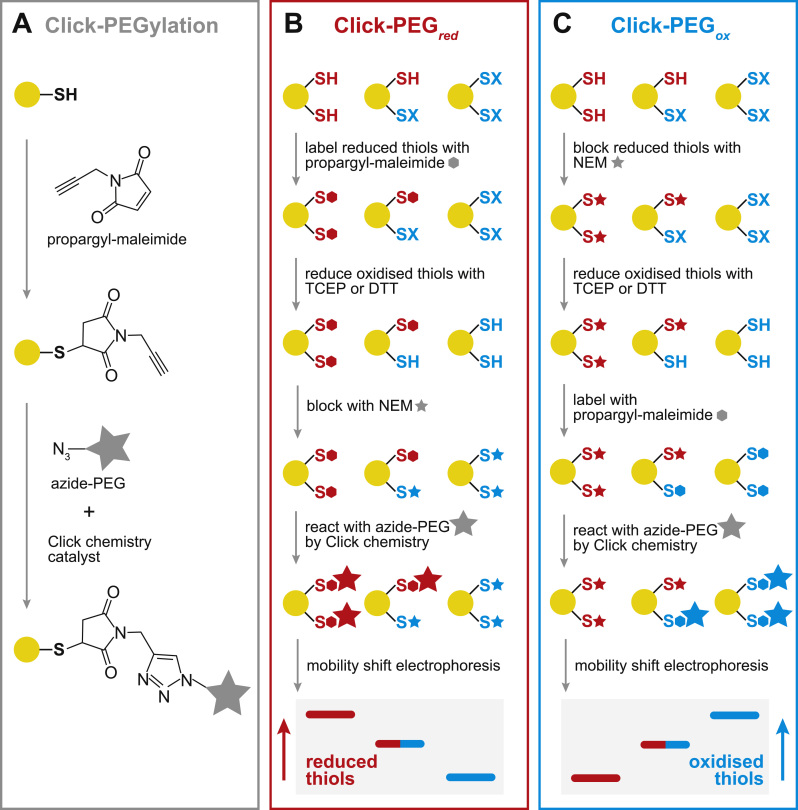
Click-PEGylation schemes. (A) Principle of the Click-PEGylation reaction. A reduced thiol is alkylated with propargyl-maleimide, then conjugated with an azide-PEG of high molecular weight (e.g. 5 kDa) using copper-catalysed Click chemistry. One of two possible regioisomers is depicted. (B) Click-PEG_*red*_ reaction to label reduced thiols. A protein with two potentially reversibly oxidisable cysteine residues is shown. For Click-PEG_*red*_, the sample is reacted with propargyl-maleimide to label reduced cysteines, which are subsequently derivatised with azide-PEG via Click chemistry. Optionally, oxidised cysteines can then be reduced *in vitro* and blocked with NEM before separation by electrophoresis. (C) Click-PEG_*ox*_ reaction to label oxidised thiols. Conversely, for ClickPEG_*ox*_, the sample is first reacted with NEM to block any reduced cysteine residues. Next, previously oxidised thiols are reduced in vitro, allowing their reaction with propargyl-maleimide and derivatisation with azide-PEG. Finally, samples are separated by electrophoresis to determine the resulting redox mobility shifts.

To address this limitation, here we have utilised Click chemistry to label redox-reactive proteins with maleimide-PEG (Fig. 1A). In Click chemistry, azide and alkyne groups are selectively reacted with each other in the presence of a Cu^1+^ catalyst [12], [13]. To label reduced cysteine residues by Click-PEGylation (Click-PEG_*red*_, Fig. 1B), reduced thiols are initially reacted with a maleimide compound containing a Click-reactive alkyne moiety, then subsequently conjugated to a large PEG molecule containing a complementary Click-reactive azide moiety, to selectively PEGylate the Click-tagged thiols. The sample can then be separated by electrophoresis, and analysed by protein staining or by Western Blotting. This basic protocol can also be altered to Click-PEGylate reversibly oxidised cysteine residues (Click-PEG_*ox*_, Fig. 1C), providing complementary information. In this case, reduced thiol groups are first blocked with *N*-ethylmaleimide (NEM), before reducing any reversibly oxidised thiols *in vitro* and subjecting these to Click-PEGylation. The major advantage of this Click-PEGylation approach is that it can be applied simply to the experimental sample, avoiding the slow and difficult direct reaction of a bulky maleimide-PEG polymer with cysteine residues. Furthermore, this method could easily be adapted to detect a specific cysteine residue modification selectively, by exploiting a particular biochemical reactivity at the labelling stage - e.g. treatment with Cu/ascorbate to identify *S*-nitrosation, dimedone for *S*-sulfenylation, or hydroxylamine for *S*-acetylation [14], [15], [16]. Finally, this Click chemistry approach to label cysteine thiol groups can be extended by substituting the azide-PEG moiety for any desired azide-containing tag to allow other modes of detection. Here we describe the development of the Click-PEGylation method and provide robust protocols for its use.

## 2 Materials and methods

### 2.1 Click-PEGylation protocol

Purified GAPDH (from rabbit muscle, Sigma G2267) or catalase (from bovine liver, Sigma C40) was reconstituted in HEND buffer (25 mM HEPES, 1 mM EGTA, 10 μM neocuproine, 100 μM DPTA and 2% w/v SDS; pH 7.4 with NaOH) at a protein concentration of 0.1 mg/mL or 0.2 mg/mL, respectively. To obtain maximally reduced and oxidised controls, samples were pre-treated with either 10 mM TCEP (tris(2-carboxyethyl)phosphine hydrochloride, Thermo Fisher Scientific 20490; pH 7.4) or 1 mM diamide (Sigma D3648; made up fresh for each experiment), respectively, and incubated at 37 °C for 30 min with gentle shaking (500 rpm, Eppendorf ThermoMixer). Reduced controls were bubbled through with argon or nitrogen gas for 30 s and sealed with parafilm prior to incubation. Afterwards, excess TCEP was removed by applying samples to a spin column (Micro Bio-Spin 6, 6000 MW limit, Tris-buffered; BioRad 732–6222 - pre-equilibrated in HEND buffer prior to use).

#### 2.1.1 Labelling of reduced cysteine residues by Click-PEG_red_

During Click-PEG_*red*_, reduced cysteine residues are PEGylated (Fig. 1B). Samples were incubated with 5 mM propargyl-maleimide (Jena Bioscience CLK-TA113 or Click Chemistry Tools TA113) at 37 °C for 30 min with agitation (1400 rpm). Excess propargyl-maleimide was then removed by passing samples through a spin column. Optionally, oxidised thiols can then be reduced *in vitro* with 10 mM TCEP, and blocked by reaction with 100 mM *N*-ethylmaleimide (NEM, Sigma E1271), followed by a spin column step to remove excess NEM.

#### 2.1.2 Labelling of oxidised cysteine residues by Click-PEG_ox_

The Click-PEG_*ox*_ protocol facilitates labelling of reversibly oxidised cysteine residues. Samples were first incubated with 100 mM NEM at 37 °C for 30 min with agitation (1400 rpm) to block reduced thiols. Excess NEM was removed by passing the samples through a pre-equilibrated spin column. The flow-through was treated with 10 mM TCEP to reduce reversibly oxidised thiols, and nitrogen (or argon) gas was bubbled through the samples for 30 s prior to incubation at 37 °C for 30 min with gentle shaking (500 rpm). Excess TCEP was removed from the samples by passing through a spin column. Samples were then reacted with 5 mM propargyl-maleimide at 37 °C for 30 min with agitation (1400 rpm), after which excess propargyl-maleimide was removed by passing samples through a spin column.

#### 2.1.3 Click-reaction

To remove chelating agents from the above Click-PEG_*red*_ and Click-PEG_*ox*_ samples that would impede the copper-catalysed Click reaction between propargyl-maleimide and azide-PEG, the protein was precipitated by adding 4 volumes of ice-cold acetone and storing the protein samples at –20 °C for a minimum of 2 h. Precipitated protein was pelleted by centrifugation for 30 min at 16,000×*g* at 4 °C and washed once with ice-cold acetone. The resulting pellet was air-dried at room temperature, then resuspended in 50 mM Tris-HCl (pH 7.4), 0.5% v/v SDS, containing 10 mM azide-PEG. The optimal PEG size will depend on the molecular weight of the protein target and the number of redox-sensitive cysteine residues. A range of azide-PEG moieties are available including: 5 kDa = azide-PEG_5000_ (methoxypolyethylene glycol azide 5000, Sigma 689475; or Creative PEGworks PLS-2024); 2 kDa = azide-PEG_2000_ (methoxypolyethylene glycol azide 2000, Sigma 689807), and 1 kDa = azide-PEG_1000_ (methoxypolyethylene glycol azide 1000, Sigma 733407).

To initiate the Click reaction, 10% v/v of freshly made catalyst components (or the Tris-HCl buffer for the ‘– catalyst’ control) were added to samples in the following order: l-ascorbic acid to a final concentration of 2.5 mM (Sigma A5960; made up fresh for each experiment), TBTA (tris[(1-benzyl-1H-1,2,3,-triazol-4-yl)methyl]amine, Sigma 678937; in 80% *t*-butanol:20% DMSO) to a final concentration of 0.1 mM, and CuSO_4_·5H_2_O (Sigma C7631; made up fresh for each experiment) to a final concentration of 1 mM. The samples were incubated at 37 °C for 1 h at 1400 rpm. The reaction was stopped by adding the appropriate volume of reducing gel loading buffer to the samples (e.g. 4X Laemmli buffer, BioRad 161–0747, supplemented with 5% v/v β-mercaptoethanol or 50–100 mM DTT; or 5X reducing sample buffer, Thermo Fisher Scientific 39000) and heating for 5 min at 95 °C, followed by brief centrifugation. Samples were either used immediately or stored at –20 °C until required for mass shift analysis by SDS-PAGE and Coomassie staining/immunoblotting.

### 2.2 Cell culture and preparation of cell lysate samples

C2C12 mouse myoblast cells (ECACC) were cultured in high glucose (4.5 g/L) Dulbecco's Modified Eagle Medium (DMEM + GlutaMAX, Thermo Fisher Scientific), supplemented with 10% v/v fetal calf serum, 100 U/mL penicillin and 100 µg/mL streptomycin. Cells were routinely passaged and maintained at sub-confluence in a humidified incubator at 5% CO_2_, 37 °C.

Cell samples were lysed in ice-cold HEND buffer containing 5 mM propargyl-maleimide (ClickPEG_*red*_ protocol) or 100 mM NEM (ClickPEG_*ox*_ protocol), incubated at 37 °C for 30 min with agitation (1400 rpm), sonicated (10×1 s; Q700 sonicator, Qsonica), centrifuged (13,000×*g* for 20 min at 4 **°**C), and the protein concentration of the resulting supernatant adjusted to 0.5 mg/mL. For maximal denaturation of complex biological samples, 6 M urea was added to the HEND buffer. The Click-PEG_*red*_ and Click-PEG_*ox*_ protocols were then performed as described above for the purified protein samples.

### 2.3 SDS PAGE and Western blotting

SDS-PAGE and Western blotting were performed according to standard laboratory protocols. Samples (~2–5 µg for purified protein; ~20 µg protein for complex biological samples) in reducing loading buffer were separated by SDS-PAGE to ensure optimal resolution for the expected range of molecular weights (typically on a 10% gel, although this will depend on the size of the target protein and the number of redox-reactive cysteine residues). Protein bands were either stained directly (e.g. with Coomassie stain for purified protein samples) or detected by Western blotting. Typically, membranes (either nitrocellulose or PVDF) were blocked for 1 h at room temperature, then incubated overnight at 4 °C with primary antibody (anti-GAPDH, rabbit polyclonal, Sigma G9545, 1:5000 for purified GAPDH experiments or 1:1000 for cell lysates; or anti-catalase, mouse monoclonal, Sigma C0979, 1:20,000). Blots were next incubated for 1 h at room temperature with secondary antibody (anti-mouse IgG HRP, polyclonal, 1:10,000, Sigma A4416; anti-rabbit IgG HRP, monoclonal, 1:10,000, Sigma A1949; or anti-rabbit IgG IRDye 800, Rockland Antibodies and Assays 611-132-003, 1:20,000). Bands were visualised as appropriate, using chemiluminescence or fluorescence. To quantify redox-dependent mobility shifts, blot images were analysed for profile plots of lanes or relative band densities (e.g. using FIJI, BioRad Image Lab, or Li-Cor Image Studio software).

## 3 Results

### 3.1 Basic Click-PEGylation protocol

The basic Click-PEGylation reaction scheme is shown in Fig. 1A. The essence of the assay is to differentially label cysteine residues that are unmodified (i.e. reduced) and those that are reversibly oxidised, with either a low molecular weight thiol alkylating agent, or with one that can be further conjugated to a high molecular weight PEG polymer. This assay can be designed to PEGylate either the reduced or oxidised thiols, described as Click-PEG_*red*_ and Click-PEG_*ox*_, respectively.

For Click-PEG_*red*_ (Fig. 1B), reduced thiol groups within a protein sample are reacted with a maleimide derivative, propargyl-maleimide, which contains an alkyne group. The stable, redox-tagged protein can then be conjugated to an azide-PEG polymer by copper-catalysed Click chemistry. The net effect is to label reduced cysteine residues with a large PEG polymer. Optionally, reversibly oxidised thiols can be reduced *in vitro* by reaction with TCEP or DTT, and blocked with the thiol alkylating agent NEM. The sample can then be separated by SDS-PAGE and the target protein visualised by protein staining or immunoblotting. Thus, in Click-PEG_*red*_, proteins with reduced cysteine residues undergo a mobility shift and appear as higher molecular weight bands, enabling the redox state of the protein to be inferred.

Conversely, the approach can be transposed to Click-PEG_*ox*_ (Fig. 1C), which detects reversibly oxidised cysteine residues. Here, reduced thiols within the sample are first blocked with NEM, then the reversibly oxidised thiols are reduced *in vitro* by reaction with TCEP or DTT, and the resulting freshly reduced thiols sequentially reacted with propargyl-maleimide and azide-PEG, enabling oxidised cysteine residues to be shifted upon PEG conjugation.

There are potential advantages to both approaches depending on the protein of interest and its inherent redox status. Most importantly, analysing a sample in parallel by Click-PEG_*red*_ and Click-PEG_*ox*_ should generate complementary information that enhances confidence in the redox state determined. For instance, some cysteines might be irreversibly oxidised, which will become evident by using both approaches side-by-side.

### 3.2 Assessing the Click-PEGylation protocols using purified GAPDH

**Fig. 2:**
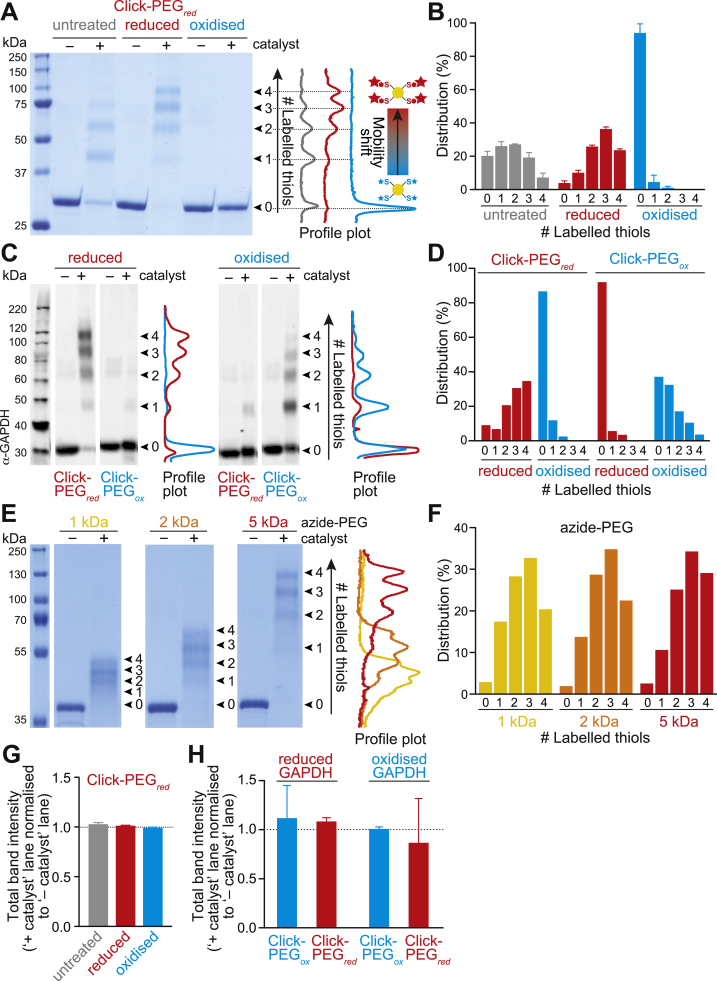
Assessment of Click-PEGylation using purified GAPDH *in vitro*. (A) Click-PEG_*red*_ of purified rabbit GAPDH, which contains 4 cysteine residues, and can therefore exist in 5 possible redox states from 0 to 4 labelled cysteine thiols. Coomassie-stained mobility shift gel of GAPDH under untreated (i.e. endogenous), reduced (10 mM TCEP), and oxidised (1 mM diamide) conditions, showing redox-dependent band shifting. Profile plots of the ‘+ catalyst’ lanes were performed in FIJI. (B) Quantification of GAPDH cysteine redox state distribution from (A). Band densitometry was performed in FIJI and expressed as a % of total band intensity per lane. Data are means±SEM of n=4 independent experiments. (C) Parallel Click-PEG_*red*_ and Click-PEG_*ox*_ of purified GAPDH under reduced (10 mM TCEP), and oxidised (1 mM diamide) conditions, detected by Western blotting, showing redox-dependent band shifting. Profile plots of the ‘+ catalyst’ lanes were performed in FIJI. (D) Quantification of GAPDH cysteine redox state distribution from (C). Band densitometry was performed in FIJI and expressed as a % of total band intensity per lane (n=1). (E) Effect of different azide-PEG sizes on the Click-PEG_*red*_ band shifting. Coomassie-stained mobility shift gel of purified GAPDH under reduced conditions (10 mM TCEP) derivatised with either 1, 2 or 5 kDa azide-PEG. Profile plots of the ‘+ catalyst’ lanes were performed in FIJI. (F) Quantification of band shifting distribution from (E). Band densitometry was performed in FIJI and expressed as a % of total band intensity per lane (n=1). (G) Assessment of sample recovery following the Click-PEG reaction. Quantification of total band intensity for the ‘+ catalyst’ lane of the Coomassie-stained gel in (A), assessed using FIJI and normalised to the ‘– catalyst’ lane as a loading control. Data are means±SEM of n=3 independent experiments. (H) Quantification of total band intensity for the ‘+ catalyst’ lane of the Western blot in (C), assessed using LiCor Image Studio software and normalised to the ‘– catalyst’ lane as a loading control. Data are means±SEM of n=3 independent experiments.

We set out to optimise and assess the potential utility of both the Click-PEG_*red*_ and Click-PEG_*ox*_ protocols using glyceraldehyde-3-phosphate dehydrogenase (GAPDH) as an exemplar protein. Initially, we utilised purified GAPDH protein from rabbit, which contains 4 cysteine residues, enabling the efficacy of the Click-PEG reactions to be directly assessed by SDS-PAGE and Coomassie protein staining (Fig. 2). For the Click reaction step, samples were split and treated in parallel with and without Click catalyst, to visualise the redox-dependent mobility shift (‘+ catalyst’). The unshifted lane (‘– catalyst’) serves as a loading control, indicating total levels of a particular target protein, which is important for quantification purposes. First, the Click-PEG_*red*_ protocol, designed to facilitate the selective labelling and shifting of reduced protein thiols, was tested on purified GAPDH reacted with propargyl-maleimide and derivatised with azide-PEG_5000_, then visualised by Coomassie staining after SDS-PAGE (Fig. 2A). We compared untreated GAPDH (i.e. reflecting the endogenous redox state), with GAPDH that was either fully reduced or oxidised *in vitro* to highlight the redox range extremes. For all conditions, GAPDH samples in the absence of Click catalyst were present only as a single band, confirming that PEGylation is fully catalyst-dependent. In samples treated with the reductant TCEP, Click-PEGylation resulted in a redox-dependent mobility shift of GAPDH relative to the untreated condition, visible as the appearance of higher molecular weight bands. Conversely, in response to pre-treatment with the thiol oxidant diamide, loss of PEGylation was observed relative to the untreated condition. This confirmed that GAPDH was as expected present in a range of redox forms with 0–4 reduced thiols groups, which were responsive to the redox environment. The redox-dependent PEGylation-induced mobility shifts can easily be visualised by plotting the densitometry profiles for each ‘+ catalyst’ lane (Fig. 2A). Individual PEGylated bands can also be quantified by densitometry, to show the relative distribution of GAPDH thiol redox states in response to the various treatments (Fig. 2B).

In addition, we confirmed that purified GAPDH in its different redox states could similarly be assessed by immunoblotting (Fig. 2C-D), obtaining comparable results to the direct protein staining (Fig. 2A-B). We also tested the efficacy of the Click-PEG_*red*_ and Click-PEG_*ox*_ protocols performed in parallel using purified GAPDH, comparing *in vitro* reduced and oxidised samples (Fig. 2C-D). As anticipated, these approaches provided complementary information on redox state. Indeed, *in vitro* reduced GAPDH displayed strong band shifting when detected by Click-PEG_*red*_ but not by Click-PEG_*ox*_, and vice versa for *in vitro* oxidised GAPDH. Note that in some cases, depending on the proximity of cysteine residues, conjugation of the target protein with PEG polymers may obstruct the epitope recognition site of some antibodies, leading to loss of immunoblot signal upon Click-PEGylation, but not in the ‘– catalyst’ control. If this occurs, alternative antibodies (particularly polyclonal) should be explored, where PEGylation will not interfere with antibody recognition of the protein.

### 3.3 Effect of azide-PEG size on redox-dependent band shifts

To test the effect of azide-PEG polymer size, we compared the Click-PEGylation response of purified GAPDH reacted with propargyl-maleimide and then conjugated to a range of PEG moieties including 1, 2, and 5 kDa (Fig. 2E). Using samples of *in vitro* reduced GAPDH to focus on maximal shifting, the densitometry profile plots illustrate how varying the PEG size results in a corresponding shifting of the bands, with 5 kDa (azide-PEG_5000_) providing the best band separation and resolution for the Click-PEGylation of GAPDH. However, regardless of the azide-PEG size selected, the resulting distribution pattern of GAPDH thiol redox state was not affected (Fig. 2F). Therefore, depending on the overall molecular weight of the candidate protein, as well as the total number of cysteine residues present, the optimal PEG size can be established (other azide-PEG sizes, e.g. 10 kDa, are also commercially available). Note that the molecular weight shifting obtained upon Click-PEGylation is not directly additive to the combined size of the PEG moieties. This is due to drag and steric hindrance for the migration of branched Click-PEGylated proteins through a gel compared to unlabelled linear proteins during electrophoresis.

Overall, we have shown, using purified GAPDH protein as a redox-responsive exemplar, that the Click-PEG_*red*_ and Click-PEG_*ox*_ protocols are useful techniques individually or in parallel to assess protein redox state by mobility shift electrophoresis. Importantly, we found no loss of sample upon PEGylation comparing total protein content between the ‘– and + catalyst’ lanes both in the case of direct protein detection by Coomassie staining (Fig. 2G) and immunoblotting (Fig. 2H), indicating that the Click-PEGylation approach generates a complete snapshot of GAPDH redox state.

### 3.4 Optimisation of the Click-PEGylation protocols

**Fig. 3:**
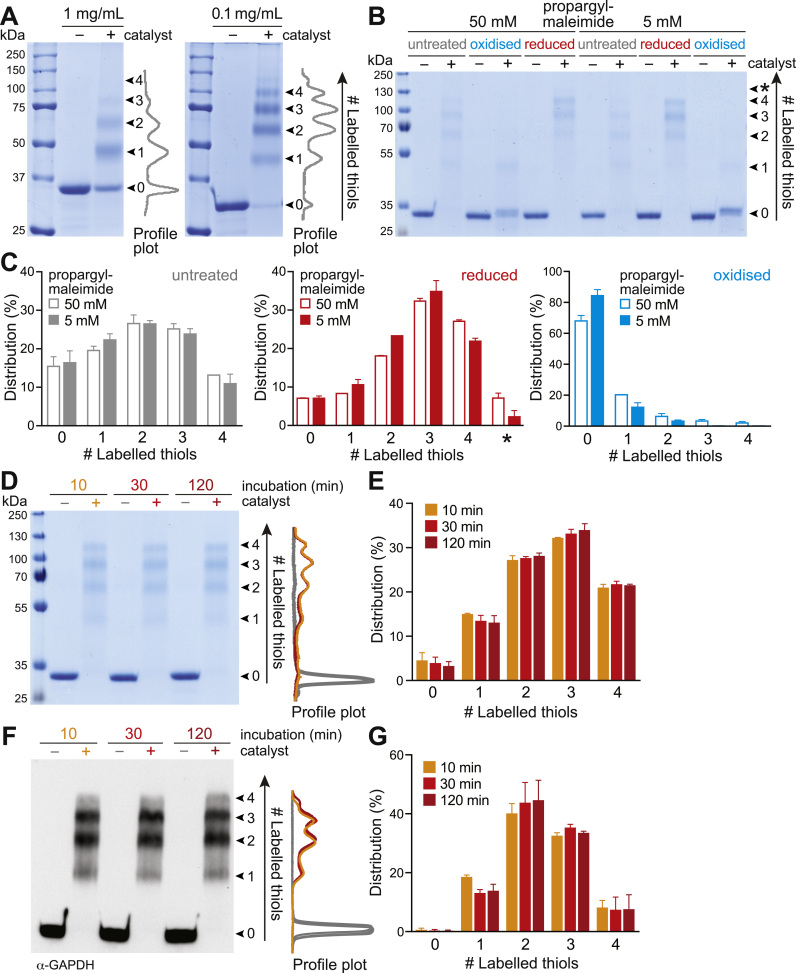
Optimisation of the Click-PEGylation technique. (A) Effect of protein concentration on labelling efficiency during the Click-PEG reaction. Coomassie-stained mobility shift gels of purified GAPDH under untreated conditions reacted by Click-PEG_*red*_, comparing starting concentrations of 1 and 0.1 mg protein/mL. Profile plots of the ‘+ catalyst’ lanes were performed in FIJI. (B) Effect of propargyl-maleimide concentration on Click-PEG labelling. Coomassie-stained mobility shift gels of purified GAPDH reacted by Click-PEG_*red*_ comparing 5 and 50 mM propargyl-maleimide, under untreated, reduced (10 mM TCEP) and oxidised (1 mM diamide) conditions. * indicates a higher molecular weight band. (C) Quantification of GAPDH thiol redox state distribution from (B). Band densitometry was performed in FIJI and expressed as a % of total band intensity per lane. Data are means ±range of n=2 independent experiments. (D) Effect of propargyl-maleimide incubation time on Click-PEG labelling. Coomassie-stained gel of purified GAPDH under reduced conditions (10 mM TCEP) reacted by Click-PEG_*red*_, comparing incubation times of 10, 30 and 120 min. Profile plots of the ‘–/+ catalyst’ lanes were performed in FIJI. (E) Quantification of GAPDH thiol redox state distribution from (D). Band densitometry was performed in FIJI and expressed as a % of total band intensity per lane. Data are means±range of n=2 independent experiments. (F) Effect of propargyl-maleimide incubation time on Click-PEG labelling. Time course as for (D), except that GAPDH redox state was assessed by Western blotting. Profile plots of the ‘–/+ catalyst’ lanes were performed in FIJI. (G) Quantification of GAPDH thiol redox state distribution from (F). Band densitometry was performed in FIJI and expressed as a % of total band intensity per lane. Data are means±range of n=2 independent experiments.

To optimise labelling conditions during the Click-PEGylation protocols, we performed a number of control experiments (Fig. 3). First, we considered sample protein concentration, and found that PEGylation efficiency was significantly improved by decreasing the starting protein concentration from 1 to 0.1 mg/mL (Fig. 3A). This is consistent with previous findings for redox proteomic sample preparation [2], and indicates the need to optimise the ratio of protein thiol content to tag compound in order to ensure complete labelling and therefore an accurate representation of protein redox status. Next, we optimised the concentration of propargyl-maleimide during the initial labelling of redox-reactive free thiols. While an excess of propargyl-maleimide at this step is desirable to ensure complete labelling, we observed that an excessive maleimide to thiol ratio led to non-specific protein labelling (Fig. 3B-C), which is corroborated by previous studies [17], [18]. Excess propargyl-maleimide (50 v. 5 mM) resulted in off-target PEGylation of residues other than cysteines during the Click-PEG_*red*_ reaction, manifested as an additional higher molecular weight band in reduced samples, as well as apparent incomplete loss of labelling in oxidised samples (Fig. 3B-C). Also, we found that preventing carry-over of excess propargyl-maleimide to the Click-labelling step was critical, by applying samples to spin columns, since any excess propargyl-maleimide may react with free azide-PEGs and therefore impair Click-PEGylation (data not shown, see the Troubleshooting Guide in Table S1). Furthermore, we optimised the duration of the propargyl-maleimide reaction step to ensure complete labelling, comparing 10, 30 and 120 min incubation times. We confirmed by both direct protein staining and immunoblotting that propargyl-maleimide labelling was complete by 10 min incubation, with no further changes at subsequent time points (Fig. 3D-G). Therefore, the majority of experiments presented in this study were performed with a 30 min propargyl-maleimide incubation. Finally, we found that varying the concentration of reducing agent, azide-PEG or catalyst from our standard Click-PEG protocol described did not alter the labelling pattern of GAPDH, indicating that GAPDH is fully reduced and Click-PEGylated (data not shown, see the Troubleshooting Guide in Table S1). Therefore any instances where fully reduced GAPDH is not maximally Click-PEGylated (i.e. shifting up to form a single higher molecular weight band indicative of 4 reduced thiols) is most likely due to irreversibly modified thiol groups that are unreactive with maleimide. Finally, we also compared different buffers for the Click Chemistry reaction (data not shown, see Troubleshooting Guide in Table S1), and found that a Tris-based buffer gave the most satisfactory results compared to HEPES-based buffers and commercially available buffers (e.g. ‘Click-iT’, Thermo Fisher Scientific) [19], [20].

### 3.5 Assessing the redox state of higher molecular weight proteins by Click-PEGylation: catalase

**Fig. 4:**
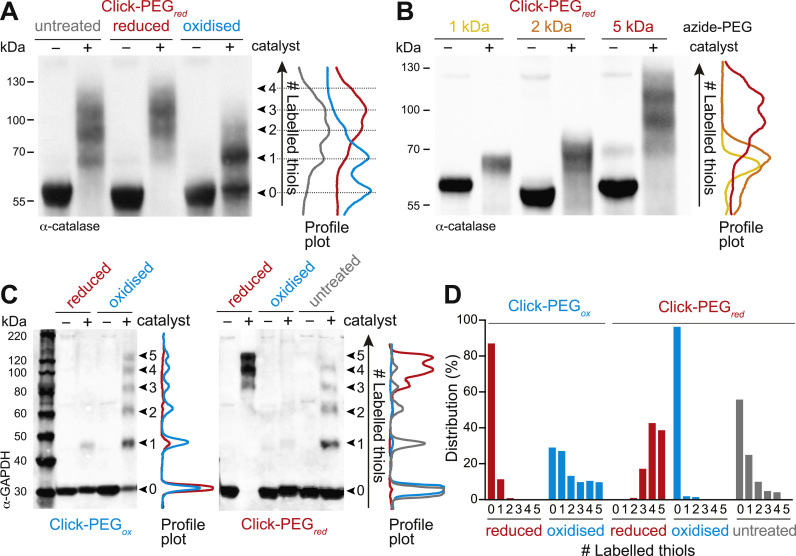
Application of Click-PEGylation to purified catalase and to endogenous GAPDH in biological samples. (A) Click-PEG_*red*_ of purified catalase under untreated, reduced (10 mM TCEP) and oxidised (1 mM diamide) conditions, detected by Western blotting. Profile plots of the ‘+ catalyst’ lanes were performed in FIJI. (B) Effect of different azide-PEG sizes on the Click-PEG_*red*_ band shifting. Western blot of purified catalase under reduced conditions (10 mM TCEP) derivatised with either 1, 2 or 5 kDa azide-PEG. Profile plots of the ‘+ catalyst’ lanes were performed in FIJI. (C) Analysis of endogenous GAPDH from cell lysates (C2C12 mouse myoblast) by Click-PEG_*ox*_ and Click-PEG_*red*_ detected by Western blotting. *In vitro* treatment of cell lysates under reduced (10 mM TCEP) and oxidised (1 mM diamide) conditions, as well as *in situ* analysis of GAPDH redox status in cell culture (untreated). (D) Quantification of band shifting in (C). Band densitometry was performed in FIJI and expressed as a % of total band intensity per lane (n=1).

So far the Click-PEGylation assay has been used effectively with purified GAPDH, which has a molecular weight of ~36 kDa. To confirm that the Click-PEGylation method is widely applicable to a broad range of proteins with differing sizes, we also tested another candidate, catalase, with a higher molecular weight (~60 kDa predicted) and 4 cysteine residues [21]. Comparable to experiments with GAPDH, we found that purified catalase was increasingly Click-PEGylated upon reduction and that this was decreased upon oxidation (Fig. 4A). Again we compared the effect of different sized azide-PEGs (1 kDa, 2 kDa, and 5 kDa) on the band shifting resolution of fully reduced catalase by Click-PEG_*red*_ (Fig. 4B). Unlike with GAPDH, we found that only azide-PEG_5000_ was able to resolve the different redox-shifted bands, sufficiently whereas the 1 and 2 kDa sizes were ineffective, as evident from the unresolved profile plots (Fig. 4B). This highlights the importance of optimising the size of the azide-PEG moiety for a given protein of interest.

### 3.6 Assessing the redox state of GAPDH by Click-PEGylation in complex biological samples

Having validated the Click-PEGylation technique with purified GAPDH and catalase as a proof-of-principle, we next wanted to demonstrate that the Click-PEGylation approach can be applied to detect redox state changes of target proteins within complex biological samples, such as cell lysates and tissue extracts. To assess endogenous redox state for a given target, the Click-PEGylation reaction is performed on the complex protein sample, and then specific detection of the redox mobility shifting for the protein of interest is achieved by immunoblotting. Therefore the redox state of multiple proteins can be assessed from the sample by parallel/successive immunoblotting using appropriate antibodies. Tagged proteins (e.g. Flag, HA) can also be assessed, using antibodies against the relevant tag.

Here, we consider the response of endogenous GAPDH in cell culture. We applied the Click-PEGylation technique to label GAPDH in mouse C2C12 myoblast lysates (Fig. 4C-D). We performed parallel Click-PEG_*red*_ and Click-PEG_*ox*_ protocols on TCEP- and diamide-treated cell lysates, and observed complementary redox-dependent band shifting for these reduced and oxidised conditions (note that mouse GAPDH contains 5 cysteine residues). We also considered untreated cell lysates, and found that endogenous GAPDH in cell culture is present in a range of redox states (Fig. 4C-D), which is consistent with reports in the literature for GAPDH *in vivo* (e.g. [2], [22]). Therefore, the Click-PEG_*red*_ and Click-PEG_*ox*_ protocols are useful techniques individually or in parallel to determine protein redox state by mobility shift electrophoresis in complex biological samples.

## 4 Conclusions

Here we describe an approach to differentially label cysteine residues according to thiol redox state, which is assessed by mobility shift electrophoresis. In Click-PEGylation, the thiol labelling step by maleimide derivatisation is separated from the PEG labelling, via a Click chemistry conjugation reaction, which gives improved flexibility over existing single-step strategies [11]. Thiol labelling, particularly of proteins in their native state, can be impaired by the bulkiness of the PEG polymers, which can limit them from reaching their target sites. For this reason, the Click-PEGylation approach - utilising a Click chemistry intermediate linking step between the labelling and the tagging - is particularly useful. Click-PEGylation can also be adapted to incorporate different tags. One could for example react samples with propargyl-maleimide and, as well as PEGylating some of them, react the remaining samples with other tags (e.g. a fluorescent label or biotin) for further enrichment, mass spectrometry or other downstream applications. Compared to other electrophoretic gel-based redox assays, thiol PEGylation has the advantage of providing the user with details about the contribution of different redox states of a protein whereas other assays, such as fluorescent labelling, generally result in a measure of overall protein oxidation.

The Click-PEGylation approach complements redox proteomics-based techniques as it is quicker and can, depending on the antibody, also facilitate a targeted assessment of candidate proteins, including proteins that are low in abundance or proteomically challenging. Here, as a proof-of-principle and to optimise the Click-PEGylation protocol, we used purified GAPDH. However, we also show that the protocol can be applied to complex samples and to thiol-containing proteins of a higher molecular weight, like catalase. Click-PEGylation would be useful to assess the effects of a range of different conditions on the redox state of candidate proteins, with interesting findings followed up by using complementary mass spectrometry-based redox methods to identify the specific cysteine residues involved. Click-PEGylation also allows the redox state of multiple targets to be interrogated from a single sample by detection with different antibodies.

In the present study, we have focused on differentially labelling reduced and reversibly oxidised thiols. To distinguish between the various possible oxidised modifications more selectively, additional steps can be incorporated into the standard Click-PEGylation protocol at the initial thiol labelling stage. For instance, *S*-nitrosated thiols could be selectively reduced using ascorbic acid, or *S*-sulfenylation specifically studied by labelling with dimedone containing an alkyne group (e.g. DYn-2 [20]). With the increasing use of Click chemistry as a selective and efficient conjugation strategy, the range of available azide-tagged probes is constantly expanding, which will allow further enhancement of the Click-PEGylation approach.
