# Supplementary material for: Click-PEGylation – A mobility shift approach to assess the redox state of cysteines in candidate proteins
Source: Free Radic Biol Med. 2017 Jul;108:374–82. doi: 10.1016/j.freeradbiomed.2017.03.037 (PMC5488967; doi:10.1016/j.freeradbiomed.2017.03.037)
Supplement: Supplementary file 1 — Supplementary material [file mmc1.docx]

**SUPPLEMENTARY MATERIAL: Table S1 - Click-PEGylation troubleshooting guide**

| **Problem** | **Possible reason** | **Solution** |
| --- | --- | --- |
| Incomplete labelling of thiols by Click-PEGylation | Unreacted propargyl-maleimide carried over to the Click-labelling step.  Inappropriate ratio of protein:label.  Some of the reagents used in the catalyst may be unstable. | Remove unreacted propargyl-maleimide before acetone precipitation by applying samples to pre-equilibrated spin columns.  Adjust the protein:label ratio, e.g. by lowering the starting protein concentration. We have found 0.1-0.5 mg protein/mL to work effectively and ensures that all labelling steps reach completeness (see Fig. 3A).  Make up ascorbic acid and CuSO_4_ stocks fresh for each experiment. |
| Non-specific protein labelling by Click-PEGylation | Inappropriate ratio of thiol:label. | An over-excess of propargyl-maleimide compared to thiol concentration may lead to non-specific labelling: lower the propargyl-maleimide concentration, but take care to maintain complete thiol labelling (Fig. 3B-C). |
| Click-PEGylated protein is inadequately or not detected by Western blotting | PEGylation may impede antibody access, depending on the cysteine distribution relative to the antibody recognition site.  The buffer during the Click chemistry reaction step needs to be optimised.  The catalyst concentration or the ascorbic acid to CuSO_4_ ratio is too high.  The SDS-PAGE transfer buffer during Western blotting needs to be adjusted. | Switch to a polyclonal antibody, or use a tagged version of the protein of interest if available. Also control for detection by quantifying total protein in the ‘–/+ catalyst’ lanes to confirm full recovery/detection (see Fig. 2G-H).  Try different buffers (e.g. HEPES v. Tris-based buffers). Limit the amount of SDS (recommended <0.5%).  Lower the overall concentration of Click catalyst components. Alternatively, adjust the ascorbic acid to CuSO_4_ ratio to generate less Cu^1+^ species.  For accurate quantification of thiol redox state distribution by band shifting, it is important to ensure complete protein transfer within the full molecular weight range of interest. Optimise the transfer conditions (e.g. wet v. semi-dry), and the buffer composition (e.g. SDS and methanol content in Tris/Glycine buffers for wet transfer). |
| Band shifts not resolved or band shape is not linear | PEG size is insufficient.  Gel % needs to be adjusted. | Optimise the azide-PEG size to improve the separation between redox state band shifts (see Fig. 2E-F and Fig. 4B).  Run samples on a more appropriate % gel. Note that gradient gels may occasionally produce ‘smiley’ bands. |
